# The effects of a limited infusion rate of fluid in the early resuscitation of sepsis on glycocalyx shedding measured by plasma syndecan-1: a randomized controlled trial

**DOI:** 10.1186/s40560-020-00515-7

**Published:** 2021-01-05

**Authors:** Jutamas Saoraya, Lipda Wongsamita, Nattachai Srisawat, Khrongwong Musikatavorn

**Affiliations:** 1 Division of Academic Affairs, Faculty of Medicine, Chulalongkorn University, Bangkok, Thailand; 2 Department of Emergency Medicine, King Chulalongkorn Memorial Hospital, The Thai Red Cross Society, 1873 Rama IV Road, Pathumwan, Bangkok, 10330 Thailand; 3 Division of Nephrology, Department of Medicine, and Critical Care Nephrology Research Unit, Faculty of Medicine, Chulalongkorn University, Bangkok, Thailand; 4 Excellent Center for Critical Care Nephrology, King Chulalongkorn Memorial Hospital, Bangkok, Thailand; 5 Academy of Science, Royal Society of Thailand, Bangkok, Thailand; 6 Department of Medicine, Faculty of Medicine, Chulalongkorn University, Bangkok, Thailand

**Keywords:** Endothelial glycocalyx, Sepsis, Resuscitation, Fluid, Syndecan-1, Emergency department

## Abstract

**Background:**

Aggressive fluid administration is recommended in the resuscitation of septic patients. However, the delivery of a rapid fluid bolus might cause harm by inducing degradation of the endothelial glycocalyx. This research aimed to examine the effects of the limited infusion rate of fluid on glycocalyx shedding as measured by syndecan-1 in patients with sepsis-induced hypoperfusion.

**Methods:**

A prospective, randomized, controlled, open-label trial was conducted between November 2018 and February 2020 in an urban academic emergency department. Patients with sepsis-induced hypoperfusion, defined as hypotension or hyperlactatemia, were randomized to receive either the standard rate (30 ml/kg/h) or limited rate (10 ml/kg/h) of fluid for the first 30 ml/kg fluid resuscitation. Subsequently, the fluid rate was adjusted according to the physician’s discretion but not more than that of the designated fluid rate for the total of 6 h. The primary outcome was differences in change of syndecan-1 levels at 6 h compared to baseline between standard and limited rate groups. Secondary outcomes included adverse events, organ failure, and 90-day mortality.

**Results:**

We included 96 patients in the intention-to-treat analysis, with 48 assigned to the standard-rate strategy and 48 to the limited-rate strategy. The median fluid volume in 6 h in the limited-rate group was 39 ml/kg (interquartile range [IQR] 35–52 ml/kg) vs. 53 ml/kg (IQR 46–64 ml/kg) in the standard-rate group (*p* < 0.001). Patients in the limited-rate group were less likely to received vasopressors (17% vs 42%; *p* = 0.007) and mechanical ventilation (20% vs 41%; *p* = 0.049) during the first 6 h. There were no significantly different changes in syndecan-1 levels at 6 h between the two groups (geometric mean ratio [GMR] in the limited-rate group, 0.82; 95% confidence interval [CI], 0.66–1.02; *p* = 0.07). There were no significant differences in adverse events, organ failure outcomes, or mortality between the two groups.

**Conclusions:**

In sepsis resuscitation, the limited rate of fluid resuscitation compared to the standard rate did not significantly reduce changes in syndecan-1 at 6 h.

**Trial registration:**

Thai Clinical Trials Registry number: TCTR20181010001. Registered 8 October 2018, http://www.clinicaltrials.in.th/index.php?tp=regtrials&menu=trialsearch&smenu=fulltext&task=search&task2=view1&id=4064

**Supplementary Information:**

The online version contains supplementary material available at 10.1186/s40560-020-00515-7.

## Background

Early and aggressive fluid resuscitation is a mainstay treatment of sepsis-induced hypoperfusion in the emergency department (ED). Fluid resuscitation is typically administered in a bolus to promptly restore mean arterial pressure (MAP) and reverse the microcirculatory derangement. According to the Surviving Sepsis Campaign (SSC) Bundle: 2018 update, 30 ml/kg of crystalloids should be initiated in the first hour for the resuscitation of sepsis-induced hypoperfusion [1]. Typically, fluid in the resuscitation phase was given rapidly, with the rate at least 500 ml over 15 min or 2000 ml/h [2]. A recent study revealed that completion of the initial 30 ml/kg fluid resuscitation within 2 h was associated with faster shock resolution and decreased sepsis mortality when compared with a slower infusion rate [3].

However, the benefit of rapid fluid bolus remains questionable. The hemodynamic effect of a crystalloid bolus in sepsis resuscitation is minimal and short-lived [4–6]. In a previous clinical study, a longer time to complete 30 ml/kg fluid bolus was not associated with increased mortality in patients with sepsis-induced hypoperfusion [7]. Moreover, treating septic shock with aggressive fluid therapy might be associated with harm (e.g., higher chance of intubation) and increased patient mortality [8–10].

Endothelial glycocalyx damage is one of the deleterious effects of rapid fluid bolus. The glycocalyx has an essential role in the regulation of vascular permeability. Damage to the glycocalyx leads to disruption of the endothelial surface layer, increases vascular permeability, and accelerates organ failure [11]. Previous studies have shown that fluid resuscitation causes hypervolemia and damages the endothelial glycocalyx [12–14]. A recent observational study reported an association between increased intravenous fluid volume and increased markers of glycocalyx degradation in septic patients, but these results were potentially biased by unmeasured confounders [15].

Therefore, we designed a randomized controlled trial to investigate the effects of a limited infusion rate of fluid administered during the early phase of sepsis resuscitation on levels of plasma syndecan-1, a biomarker of glycocalyx damage, compared to those of the standard fluid resuscitation rate. We hypothesized that the limited rate of fluid resuscitation would mitigate glycocalyx damages in septic patients.

## Methods

### Study design and settings

This open-label, investigator-initiated, parallel-group study with equal randomization (1:1) was conducted in an urban, academic ED. The ED has an annual census of 80,000 visits and stands in a 1500-bed, university-affiliated tertiary care hospital. This study was approved by the Institutional Review Board, Faculty of Medicine, Chulalongkorn University (IRB No. 431/61) and was registered with the Thai Clinical Trials Registry (TCTR20181010001). This trial is reported in accordance with the Consolidated Standards of Reporting Trials (CONSORT) guidelines.

### Participants

Participants were recruited between November 2018 and February 2020. All adults aged 18 years or over presenting to the ED with suspected sepsis-induced hypoperfusion, were eligible for inclusion. Patients were defined as presumed sepsis if they had suspected infection with a quick sequential organ failure assessment (qSOFA) of ≥ 2 according to the sepsis-3 definition [16]. Hypoperfusion was defined as a systolic blood pressure (SBP) < 90 mmHg, mean arterial pressure (MAP) < 65 mmHg, or blood lactate ≥ 4 mmol/L. The lactate cut-off was revised to ≥ 2 mmol/L from April 2019 due to a slow recruitment rate after enrolling 20 participants. Participants were excluded if they met any of the following criteria: (1) received more than 500 ml resuscitation fluid; (2) had SBP < 70 mmHg; (3) had a suspected other main cause of hypoperfusion (obstructive, cardiogenic, hypovolemic, such as gastrointestinal hemorrhage); (4) had concurrent acute heart failure or known left ventricular ejection fraction (LVEF) less than 40% or severely depressed LVEF by eyeballing point-of-care ultrasound (POCUS )[17]; (5) had end-stage renal disease (ESRD) with chronic renal replacement therapy (RRT); (6) had a suspected infection from dengue virus, malaria, Leptospira, and Rickettsia; (7) had the potential need for immediate surgery within 6 h; (8) had a body mass index (BMI) ≥ 30 kg/m^2^; (9) had concurrent acute traumatic brain injury; (10) had a do-not-attempt-resuscitation (DNAR) order status; (11) were transferred from another hospital; and (12) were pregnant.

### Randomization

While eligible patients were identified, patients or their legal representatives were approached by the investigators to provide information about the trial. Written informed consent was obtained before for trial participation. A participant was randomized into either group at a 1:1 ratio using computer generated-block randomization, with blocks of varying sizes of 4, 6, and 8 prepared by an investigator without clinical involvement in the trial. Allocations were concealed in opaque, sealed envelopes and were opened after the informed consent was obtained. Participants were randomized into either the standard infusion rate group or the limited infusion rate group.

### Study interventions

Lactated Ringer’s solution (LRS) was used as an initial resuscitation fluid in both groups. For the first 30 ml/kg fluid bolus, the standard-rate group was set at a rate of 30 ml/kg/h or a maximum rate of 2000 ml/h, while the limited-rate group was assigned a rate of 10 ml/kg/h. MAP was monitored and recorded every 5 min using non-invasive blood pressure monitoring until target blood pressure was achieved (defined as MAP > 65 mmHg for at least three consecutive measurements). If the target blood pressure was not achieved within 15 min, norepinephrine was peripherally administered at a concentration of 4 mg diluted in 250 ml at a starting rate of 5 ml/h (= 1.3 μg/min) and was titrated to keep MAP > 65 mmHg. After completion of the designated fluid protocol, further fluid resuscitation rate and amounts were administered according to the physician’s discretion with a rate that did not exceed that of the designated groups for the total duration of 6 h. The use of POCUS to assist decision-making in resuscitation was mandated in every case and was performed by trained emergency medicine residents or attending physicians. The decision to insert a central venous catheter, arterial catheter, or to use corticosteroids depended on the clinician’s judgment. All patients received the standard sepsis treatment as recommended in the SSC guideline 2016 and 2018 bundle update.

During the 6-h intervention, if participants exhibited signs of fluid overload, including crepitation of lungs, SpO2 decrease > 3% or respiratory rate increase > 5/min or encountered refractory hypotension despite optimizing vasopressors, or other specific reasons of the treating physicians, the protocol was terminated, and the reasons were recorded. The physician could adjust the treatment based on the patient’s safety, such as prescribing diuretics for fluid overload or increasing the rate of fluid resuscitation in patients with persistent hypoperfusion.

### Data collection and follow-up

At baseline, after the enrollment, we collected and recorded patient characteristics, vital signs, and laboratory tests, including blood lactate, N-terminal pro-b-type natriuretic peptide (NT-proBNP), and syndecan-1 levels. Qualitative LVEF estimations by POCUS were stratified according to the four-point cardiac rating scale: severely depressed, moderately depressed, normal, and increased LVEF [17]. Since we excluded the patients with severely depressed LVEF, we categorized the LVEF of the eligible patients into “normal to increased LVEF” and “moderately depressed LVEF.” Lactate and syndecan-1 levels were measured again at 6 h. We followed all patients until their hospital discharge for 90 days to determine the clinical outcomes. If patients were discharged before 90 days, we called the patients or their representatives by phone to determine mortality outcomes. Data were collected on paper case report forms by the investigators and entered into a REDCap software database hosted at the Faculty of Medicine, Chulalongkorn University [18].

### Outcomes

The primary outcome was differences in change of syndecan-1 levels at 6 h compared to baseline between standard-rate and limited-rate groups. Secondary outcomes were proportions of patients with MAP ≥ 65 mmHg at 1 h and 6 h, 6-h lactate clearance, PaO_2_/FiO_2_ (P/F) ratio at 6 h, fluid input and fluid balance at 24 and 72 h, days alive and free of vasopressor support, mechanical ventilation or RRT up to 28 days, hospital length-of-stay (LOS), and 28-day and 90-day all-cause mortality. The 6-h lactate clearance was calculated by subtracting the lactate level at 6 h from the initial lactate level and divided by the initial lactate level (i.e., [(Initial lactate–lactate at hour 6)/Initial lactate] × 100%). Specified adverse events were monitored during the 6-h intervention period, which included cardiogenic pulmonary edema, new arrhythmia, and the incidence of norepinephrine extravasation. We also recorded protocol adherence and reasons for protocol termination. Serious adverse events were reported to the ethics committee.

### Biomarker assays

Blood samples were collected at enrollment and 6 h later to determine plasma syndecan-1 levels. Samples were collected into ethylenediaminetetraacetic acid (EDTA) tubes and stored in a refrigerator before centrifugation followed by storage at – 80 °C. Syndecan-1 levels were measured using commercial enzyme-linked immunosorbent assay (ELISA) kits (Abcam, Cambridge, MA, USA). NT-proBNP levels were measured using electrochemiluminescence immunoassay analysis (Roche Diagnostics, Mannheim, Germany)

### Sample size calculation

According to earlier research, the standard deviation (SD) of syndecan-1 in septic patients is 109 ng/ml [19]. Therefore, a sample size of 98 patients would have a power of 90% to detect a reduction of 81 ng/ml in the limited-rate group, allowing for a dropout rate of 20%, with a two-sided alpha level of 0.05.

### Statistical analysis

All analyses were performed according to the intention-to-treat principle. Continuous data are reported as the means with SD or medians with interquartile ranges (IQR), depending on the distribution after normality assessment by visual inspection. Due to highly skewed data, syndecan-1 levels were log-transformed to generate normal distributions and are reported as geometric means with 95% confidence intervals. Categorical data are reported as proportions. A Wilcoxon signed-rank test was used to analyze the change in syndecan-1 from baseline to 6 h. The primary outcome (the differences in change of syndecan-1 level at 6 h compared with that of the baseline between the two groups) was analyzed using linear regression and is reported as a geometric mean ratio (GMR). Secondary outcomes were analyzed with independent *t* test, chi-square test, Fisher’s exact test, or Wilcoxon rank sum test, depending on the types of data. We did not impute missing data. However, the numbers of observations in the analysis are reported. Secondary analysis for the primary outcome included an adjusted analysis for baseline hemodynamics and the concurrent use of vasopressors. We also tested for interactions between the intervention and prespecified subgroups (baseline syndecan-1, NT-proBNP, lactate level, and Acute Physiology and Chronic Health Evaluation (APACHE) II score). All analyses were performed using STATA version 16 (College Station, TX, USA). Statistical significance was defined as *p* < 0.05.

## Results

### Participants

**Fig. 1:**
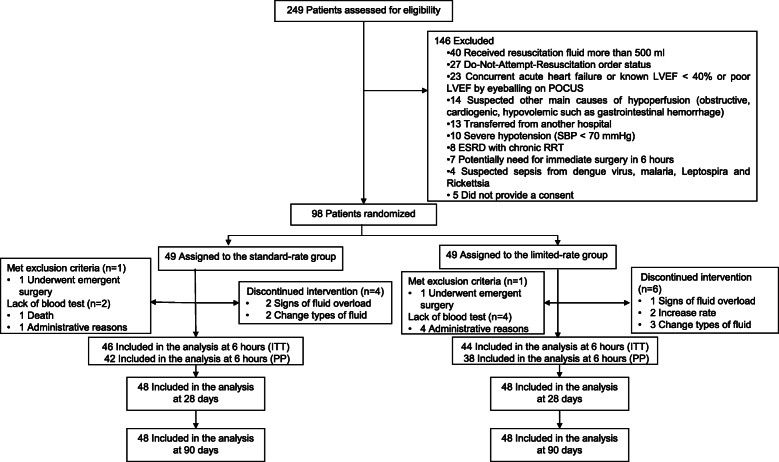
Flow diagram of enrollment, intervention allocation, follow-up, and data analysis. LVEF: left ventricular ejection fraction; POCUS: point-of-care-ultrasound; SBP: systolic blood pressure; ESRD: end-stage renal disease; RRT: renal replacement therapy; ITT: intention-to-treat; PP: per protocol

From November 2018 to February 2020, 249 patients were screened for eligibility, 146 patients met exclusion criteria, and 5 patients refused to participate in the trial. Ninety-eight patients were randomized to either the standard-rate or the limited-rate group. One patient in each group was excluded from the analysis because they met the exclusion criteria of undergoing emergency surgery. Regarding the primary outcome, syndecan-1 results were missing in two cases of the standard-rate groups and in four cases of the limited infusion rate groups due to administrative reasons and loss to follow-up. In summary, 46 and 44 participants were analyzed for the primary outcome in the standard and limited infusion rate groups, respectively. Forty-eight patients per group were analyzed regarding all other analyses not related to the syndecan-1 test. The patient flow diagram is shown in Fig. 1. The baseline characteristics of patients in both groups are comparable, but patients in the limited-rate group exhibited greater hemodynamic stability in general and had a higher prevalence of previous systemic steroid use (Table 1).

**Table 1:** Baseline Characteristics of the participants

	Standard rate (*n* = 48)	Limited rate (*n* = 48)
Age (years)	72 (16)	70(18)
Sex (female)	18 (38%)	19(40%)
Body weight (kg)	49.3 (7.9)	54.8 (11.8)
Charlson comorbidity index	5 (4, 6.5)	5 (3, 7)
Comorbidities		
Cerebrovascular disease	30 (63%)	23 (48%)
Diabetes mellitus	21 (44%)	20 (42%)
Malignancy	15 (31%)	18 (38%)
Ischemic heart disease	3 (6%)	8 (17%)
Chronic kidney disease	1 (2%)	5 (10%)
Hypoperfusion defined by		
Lactate ≥ 2 mmol/L	44 (90%)	42 (86%)
Hemodynamic instability	23 (48%)	13 (27%)
Systolic blood pressure (mmHg)	105.9 (32.8)	114.3 (29.8)
Diastolic blood pressure (mmHg)	58.4 (20.2)	66.5 (17.7)
Mean arterial pressure (mmHg)	74.6 (22.7)	82.3 (19.9)
Body temperature (degree Celsius)	38.5 (1.3)	38.1 (1.2)
Heart rate (/min)	117.8 (28.1)	119.6 (24.3)
Respiratory rate (/min)	24.3 (7.1)	24.0 (6.3)
Ambient air pulse oximetry (%)	90.2 (12.0)	93.3 (7.7)
Currently use systemic steroid	5 (10%)	12 (25%)
APACHE II	18.0 (13.0, 24.5)	15.5 (11.0, 20.0)
SOFA	5(2,6)	4(2,5)
Sepsis*	43 (90%)	43 (90%)
Septic shock*	11 (23%)	6 (13%)
Normal to increased LVEF^†^	44 (92%)	41 (85%)
Lactate (mmol/L)	4.9(3.2)	4.4(2.4)
Baseline NT-proBNP (pg/ml)^‡^	950.7 (435.5, 1946)	1188.5 (366, 2495.5)
P/F ratio at baseline (mmHg)	364.9 (174.0)	328.7 (134.3)
Intravenous fluid before randomization (ml)		
None	40 (83%)	39 (79%)
200	5 (10%)	5 (10%)
201–500	3 (6%)	5 (10%)
Site of infection		
Respiratory tract	23 (48%)	19 (40%)
Urinary tract	10 (21%)	10 (21%)
Intraabdominal	10 (21%)	10 (21%)
Bloodstream	2 (4%)	1 (2%)
Central nervous system	0	1 (2%)
Other/unknown	3 (6%)	7 (15%)
Baseline syndecan-1 level (ng/ml)^§^		
Median (Q1, Q3)	205 (136, 378)	222(126, 759)
Geometric mean (95% CI)	258 (179–373)	312 (217–451)

Data indicate the mean (SD), median (Q1, Q3), or *n* (%) unless otherwise stated

*APACHE* Acute Physiology and Chronic Health Evaluation, *SOFA* Sequential Organ Failure Assessment, *LVEF* left ventricular ejection fraction

*According to the sepsis-3 definition [16].

^†^According to the qualitative LVEF estimations [17].

^‡^Six data points are missing in the standard-rate group and four are missing in the limited-rate group due to administrative reasons

^§^One data point is missing in the limited infusion rate group due to administrative reasons

### Treatments during the 6-h intervention period

During the intervention period, the fluid administered in the limited-rate group was less than that of the standard-rate group (39 ml/kg IQR 35–52 ml/kg vs. 53 ml/kg IQR 46–64 ml/kg; *p* < 0.001). Patients in the limited-rate group were less likely to received vasopressors (17% vs. 42%; *p* = 0.007) compared to the standard-rate group. There was no difference in vasopressor dose between the groups. The use of mechanical ventilation was less frequent in the limited-rate group than in the standard-rate group (23% vs. 42%: *p* = 0.049). The use of corticosteroids was comparable in both groups (8% vs. 10% *p* = 0.73), and there was no difference in the use of albumin or time to antibiotics (Table 2). The hemodynamic data of the patients during the intervention were showed in Supplementary Table S1. The hourly fluid administration, vasopressor, and mechanical ventilation were depicted in Supplementary Figure S1, S2, and 3, respectively.

**Table 2:** Treatments during the 6-h intervention period

	Standard rate (*n* = 48)	Limited rate (*n* = 48)	*p* value
Vasopressor use	20 (42%)	8 (17%)	0.007
Mechanical ventilation	20 (42%)	11 (23%)	0.049
-Started after enrollment	16 (33%)	9 (19%)	0.10
Steroid use	5 (10%)	4 (8%)	0.73
Albumin use	2(4%)	2(4%)	> 0.99
Time to antibiotics from triage (min)	42 (30.5, 57.5)	49 (39, 66)	0.06

Data are *n* (%) and median (Q1, Q3)

### Primary outcome

**Fig. 2:**
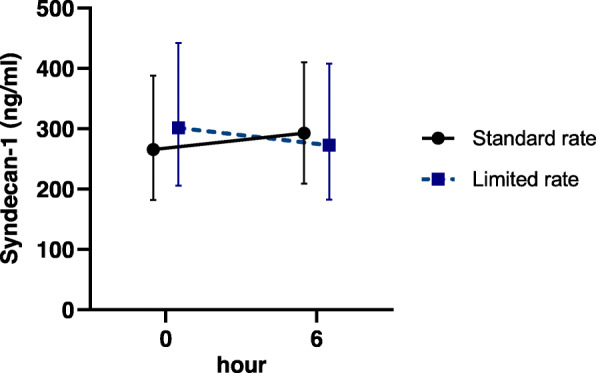
Changes in syndecan-1 levels from baseline to 6 h. (Data are presented as the geometric mean and error bars represent 95% confidence interval)

The geometric means of syndecan-1 in the standard-rate (*n* = 46) and limited-rate (*n* = 44) groups were 265 ng/ml (95% CI 182–388 ng/ml) and 301 (95% CI 206–442 ng/ml) at baseline and 293 ng/ml (95% CI 209–410 ng/ml) and 273 (95% CI 183–408 ng/ml) at 6 h, respectively. There was no significant difference in changes of syndecan-1 level at 6 h (GMR in the limited-rate group, 0.82; 95% CI 0.66–1.02; *p* = 0.07) (Fig. 2). When the data were adjusted for differences in baseline and treatment (hemodynamic status and vasopressor use within 6-h period), the difference remained insignificant (GMR in the limited-rate group, 0.80; 95% CI 0.64–1.00; *p* = 0.05). According to the per-protocol analysis (42 patients in the standard-rate and 38 patients in the limited-rate group), there was no difference between the groups (GMR in the limited-rate group, 0.84 95% CI (0.66–1.06; *p* = 0.07).

### Changes of syndecan-1 level within groups

The median syndecan-1 levels in the standard-rate group were 205 ng/ml (IQR 136–378 ng/ml) and 220 ng/ml (IQR 157–519 ng/ml) at baseline and 6 h, respectively. The median syndecan-1 levels in the limited-rate group were 221 ng/ml (IQR 127–759 ng/ml) and 198 ng/ml (IQR 106–487 ng/ml) at baseline and 6 h, respectively. During the 6-h intervention period, there was no significant different change of syndecan-1 level within the standard-rate and limited rate groups (*p* = 0.23 and 0.77, respectively).

### Protocol adherence

Protocol adherence was high in both groups since termination of the protocol occurred in only six patients (12%) in the limited-rate group and in five patients (10%) in the standard-rate group. The reasons for protocol termination were the physicians’ decision to change the type of intravenous fluid, the patients exhibited signs of fluid overload, and physicians’ decision to increase the rate of intravenous fluid (Fig. 1).

### Adverse events during intervention

There was one adverse event of cardiogenic pulmonary edema in the standard-rate group and two in the limited-rate group. There were no new arrhythmias or extravasation of peripherally administered vasopressors in either group. In the standard-rate group, one participant died during the intervention period due to life-threatening massive hemoptysis. This fatal event was reviewed and reported to the ethics committee and was considered unrelated to the intervention.

### Secondary outcomes

Regarding physiological parameters, there were no significantly different changes in 6-h lactate clearance, the proportion of patients with MAP ≥ 65 mmHg at 1 h and 6 h, or the P/F ratio at 6 h. Fluid input per body weight at 24 h was lower in the limited-rate group, but at 72 h, the volume of fluid used was comparable in both groups. There was no difference between the groups with respect to fluid balance at 24 or 72 h, organ failure-free days or hospital length of stay. The 90-day mortality was 18.8% and 31.3% in the limited-rate group and standard-rate group, respectively (relative risk in the limited-rate group 0.60 (95% CI 0.29–1.24; *p* = 0.16). The data are summarized in Table 3.

**Table 3:** Secondary outcomes

	Standard rate (*n* = 48)	Limited rate (*n* = 48)	Point estimates (95% CI)*	*p* value
6-h lactate clearance (%)	(*n* = 46) 26.8 (39.8)	(*n* = 46) 26.4 (38.1)	Mean difference − 0.5% (− 16 to 15.6%)	0.95
Patients with MAP ≥ 65 mmHg at 1 h	36/47 (77%)	42/47 (89%)	RR 1.17(0.97–1.41)	0.049
Patients with MAP ≥ 65 mmHg at 6 h	43/47 (92%)	43/48 (90%)	RR 0.98(0.86–1.12)	0.73
P/F ratio at 6 h (mmHg)	(*n* = 46) 337 (178)	(*n* = 46) 363 (159)	Mean difference 26( − 44 to 96)	> 0.99
Fluid input in 6 h (ml)	(*n* = 47) 2600 (2100, 3489)	(*n* = 48) 2238(1898, 2488)		0.003
Fluid input per body weight in 6 h (ml/kg)	(*n* = 47) 53 (46, 64)	(*n* = 48) 39 (35, 52)		< 0.001
Fluid input per body weight in 24 h (ml/kg)	(*n* = 40) 115 (86, 146)	(*n* = 45) 88 (63, 111)		0.02
Fluid balance in 24 h (ml)	(*n* = 40) 3758 (1237, 4975)	(*n* = 43) 2896 (1520, 4535)		0.68
Fluid input per body weight in 72 h (ml/kg)	(*n* = 37) 175 (124, 220)	(*n* = 37) 150 (108, 229)		0.70
Fluid balance in 72 h (ml)	(*n* = 37) 3140 (377, 5524)	(*n* = 36) 4100 (2636, 7090)		0.13
Requirement for vasopressors	27/42 (64%)	20/46 (43%)	RR 0.68 (0.45–1.01)	0.05
Days alive and free from vasopressors up to 28 days	(*n* = 44) 26 (0, 28)	(*n* = 46) 27.5 (22, 28)		0.13
Requirement for mechanical ventilation	21/42 (50%)	19/46 (41%)	RR 0.83 (0.52–1.31)	0.41
Days alive and free from mechanical ventilation up to 28 days	(*n* = 44) 27.5 (0, 28)	(*n* = 46) 27 (9, 28)		0.91
Requirement for new RRT	4/42 (10%)	6/46 (13%)	RR 1.34 (0.41–4.52)	0.74
Days alive and free from RRT up to 28 days	(*n* = 44) 28 (0, 28)	(*n* = 46) 28 (21, 28)		0.60
Days alive and free from organ failure up to 28 days	(*n* = 44) 25 (0, 27.5)	(*n* = 46) 26 (9, 28)		0.37
Hospital LOS (day)	6 (5, 14)	11 (3.5, 25)		0.23
28-day mortality	12/48 (25%)	8/48 (17%)	RR 0.67 (0.30–1.48)	0.32
90-day mortality	15/48 (31%)	9/48 (19%)	RR 0.60 (0.29–1.24)	0.16

Data are mean (SD), *n*/total *n* (%) and median (Q1, Q3)

*MAP* mean arterial pressure, *P/F* PaO_2_/FiO_2_, *RR* relative risk, *RRT* renal replacement therapy, *LOS* length-of-stay

*Point estimates are for the limited-rate group compared to the standard-rate group

### Subgroup analysis

**Fig. 3:**
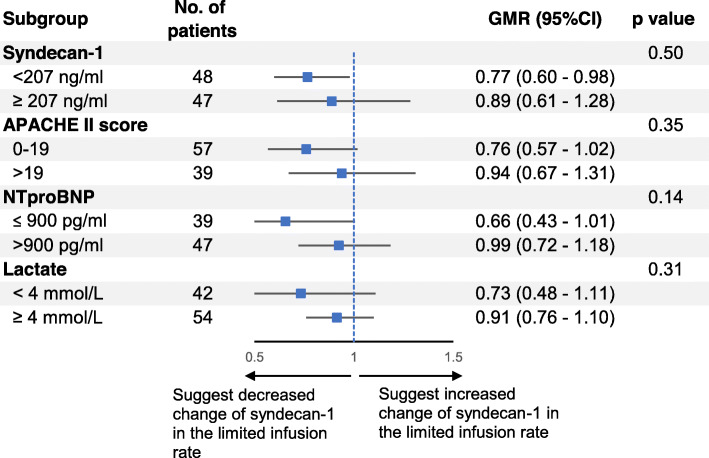
Prespecified subgroup analysis. *p* value for the interaction. (GMR: geometric mean ratio; CI: confidence interval; APACHE: Acute Physiology and Chronic Health Evaluation; NT-proBNP: N-terminal pro-b-type natriuretic peptide)

No significant difference was observed regarding the effect of limited rate according to the pre-specified subgroups, baseline syndecan-1 levels, NT-proBNP, lactate, or APACHE II score (*p* = 0.14 to 0.50 for interaction) (Fig. 3).

## Discussion

In this randomized controlled trial examining resuscitating patients with sepsis-induced hypoperfusion in the ED, the limited rate of fluid resuscitation compared to the standard rate did not significantly reduce changes in syndecan-1 at 6 h. However, reduced 6-h and 24-h fluid input volumes were observed in the limited-rate group compared to the standard-rate strategy. There was no significant difference in organ failure outcomes, adverse events, or mortality rate between the two groups.

Previous studies have reported an association between hypervolemia from rapid fluid administration and glycocalyx shedding as measured by syndecan-1 [13, 14, 20]. In an animal model of sepsis, rapid fluid administration (30 ml/kg/h) resulted in increased syndecan-1 shedding compared to the slower rate (10 ml/kg/h) [20]. In humans, increased syndecan-1 levels were detected after rapid fluid bolus in 15 min [13]. Higher levels of syndecan-1 were observed after fluid bolus in healthy preoperative patients, concurrently with higher levels of atrial natriuretic peptide (ANP) [14]. Released in response to hypervolemia, the peptide hormone ANP, and brain natriuretic peptide (BNP) were found to have in vivo activity with respect to glycocalyx shedding [21]. Moreover, rapid fluid bolus administration could lead to shear stress that directly activates secretion of matrix metalloproteinases from endothelial cells and stimulates glycocalyx shedding [22]. Though a limited-rate strategy may mitigate transient hypervolemia and shear stress from fluid administration, our study did not show a significant reduction of changes in syndecan-1 between different fluid resuscitation strategies. This finding could be explained by heterogeneity of the septic patients, which resulted in differences in patient characteristics in this small, randomized controlled trial since destruction of the endothelial glycocalyx can result from various factors, such as the inflammation, hypoxia, or vasopressor administration [19, 23, 24]. In our study, after adjusting for differences in hemodynamic instability and vasopressor administration, the effect of the limited-rate fluid strategy on pre- and post-treatment reduction in syndecan-1 levels was more pronounced but still insignificant.

Interestingly, in the standard-rate group, the use of vasopressors was more frequent than in the limited-rate group. This could partially be explained by imbalanced baseline characteristics of the participants: there were more patients with hemodynamic instability in the standard-rate group. Moreover, the proportion of mechanically ventilated patients at enrollment in the standard-rate group was higher than that in the limited-rate group (Supplementary Figure S3). This might also explain the increased use of vasopressors in the standard-rate group since mechanical ventilation potentially induced hemodynamic instability in preload-dependent patients [25]. However, previous studies also potentially provide a partial explanation from evidence of the inefficacy of rapid fluid bolus administration. In a volume kinetics study in human volunteers, the fraction of infused crystalloid that remained in the plasma was higher in response to a lower rate of infusion [26]. Another study found that cardiac output increased by 0.02 L/min in the slower fluid bolus (rate 500 ml/h) compared to the rapid fluid bolus (rate 2000 ml/h). The effect returned to baseline after infusion was complete [27].

Our study demonstrated that the limited-rate strategy led to a reduction in fluid volume used at 6 and 24 h without significant adverse events or any difference in clinical outcomes. However, this study was not adequately powered to detect differences and thus should be considered exploratory. A significant difference in clinical outcomes was not demonstrated in previous pilot studies of limited volume fluid resuscitation. In a pilot randomized study in an intensive care setting, patients with septic shock treated with the restrictive fluid approach received less fluid during the initial 5 days than those with treated with the liberal strategy (absolute difference − 1.2 L; 95% CI − 2.0 to − 0.4 L), and there was a signal towards mitigating kidney injury in restrictive fluid approach [28]. In the ED setting, implementation of the limited volume of resuscitation coupled with early vasopressor use was feasible and was associated with a decreased amount of fluid during the initial phase of resuscitation [29]. A large study that comparing the clinical outcomes of various fluid resuscitation approaches is currently being conducted and is potentially powered to determine their effects on relevant outcomes [30].

To our knowledge, this study is the first randomized trial comparing different fluid rate strategies during the very early phase of resuscitation in septic patients in the emergency department. The type of resuscitation fluid was controlled provided that different fluid types were associated with different magnitude of glycocalyx damage [31]. However, there are several notable limitations. First, the investigators, healthcare providers, and patients were not blinded to the study procedures; therefore, potential biases may affect recognition and treatment in open-label trials. However, we measured objective outcomes that are less susceptible to misclassification. Second, despite the appropriate randomization method, participants between the groups exhibited distinct different baseline characteristics. An adjusted analysis was performed to mitigate this disparity. Third, assessment of glycocalyx integrity with direct visualization (e.g., intravital microscopy) or using mass spectrometry potentially yields more accurate results than does the detection of plasma syndecan-1 by ELISA methods. However, measuring syndecan-1 levels is much more clinically practical. Syndecan-1 is negatively correlated with changes in glycocalyx thickness and positively correlated with changes in microvascular permeability [32]. Furthermore, syndecan-1 was extensively studied regarding correlations with clinical outcome [11]. Higher levels of syndecan-1 are associated with organ failure and mortality in septic patients [33, 34]. Since the dispersion of syndecan-1 levels are too large compared to effect size, larger studies with more participants are needed to potentially detect the differences in the magnitude of glycocalyx shedding and to highlight the effects of different fluid strategies on important clinical outcomes. Lastly, the infusion rate of fluid administration is definitely associated with its total volume. In our study, patients in the limited-rate group received lower fluid volume than that of the other. Although we aimed to investigate the effect of rate on the marker of glycocalyx shedding, the outcomes could have been affected by the difference in fluid volume.

## Conclusions

In patients with sepsis-induced hypoperfusion, the administration of resuscitative fluid with a limited fluid infusion rate did not significantly reduce changes in syndecan-1 at 6 h, but it reduced the volume used during the early resuscitation compared to patients resuscitated using the standard-rate approach.

## Supplementary Information

**Additional file 1: Table S1.** Hemodynamic data of the patients during the intervention. **Figure S1.** Mean hourly intravenous fluid volume per body weight (ml/kg) during the 6-hour intervention period. The error bars represent the standard deviation. **Figure S2.** Cumulative number of patients with the need of vasopressors during the 6-hour intervention period. **Figure S3.** Cumulative number of mechanically-ventilated patients during the 6-hour intervention period.

## Data Availability

The datasets used and/or analyzed during the current study are available from the corresponding author on reasonable request.

## Abbreviations

ANP: Atrial natriuretic peptide
APACHE: Acute Physiology and Chronic Health Evaluation
BMI: Body mass index
BNP: Brain natriuretic peptide
CI: Confidence interval
CONSORT: Consolidated Standards of Reporting Trials
DNAR: Do-not-attempt-resuscitation
ED: Emergency department
EDTA: Ethylenediaminetetraacetic acid
ELISA: Enzyme-linked immunosorbent assay
ESRD: End-stage renal disease
GMR: Geometric mean ratio
IQR: Interquartile range
IRB: Institutional Review Board
LOS: length-of-stay
LRS: Lactated Ringer’s solution
LVEF: Left ventricular ejection fraction
MAP: Mean arterial pressure
NT-proBNP: N-terminal pro-b-type natriuretic peptide
P/F: PaO2/FiO2
POCUS: Point-of-care ultrasound
qSOFA: Quick sequential organ failure assessment
RRT: Renal replacement therapy
SBP: Systolic blood pressure
SD: Standard deviation
SSC: Surviving Sepsis Campaign
TCTR: Thai Clinical Trials Registry
