# Supplementary material for: The effects of a limited infusion rate of fluid in the early resuscitation of sepsis on glycocalyx shedding measured by plasma syndecan-1: a randomized controlled trial
Source: J Intensive Care. 2021 Jan 5;9:1. doi: 10.1186/s40560-020-00515-7 (PMC7784279; doi:10.1186/s40560-020-00515-7)

**Supplementary material**

**Table S1** Hemodynamic data of the patients during the intervention.

|  | Standard rate (n= 48) | | | Limited rate (n = 48) | | |
| --- | --- | --- | --- | --- | --- | --- |
|  | Hour 0 | Hour 1 | Hour 6 | Hour 0 | Hour 1 | Hour 6 |
| SBP (mmHg) | 105.9 (32.8) | 115.9 (26.1) | 113.0 (23.6) | 114.3 (29.8) | 117.4 (27.2) | 116.8 (23.4) |
| DBP (mmHg) | 58.4 (20.2) | 62.4 (16.9) | 65.3 (15.3) | 66.5 (17.7) * | 66.7 (16) | 67.4 (13.2) |
| MAP (mmHg) | 74.6 (22.7) | 79.4 (19.2) | 81.0 (16.7) | 82.3 (19.9) | 83.6 (18.5) | 82.8 (15.9) |
| Heart rate (/min) | 117.8 (28.1) | 109.4 (23.3) | 99.2 (18.1) | 119.6 (24.3) | 108.0 (23.2) | 103.0 (18.9) |
| Respiratory rate (/min) | 22.0  (20.0, 27.0) | 20.0  (20.0, 22.0) | 20.0  (18.0, 20.0) | 22.0  (20.0, 25.0) | 20.0  (20.0, 22.0) | 20.0  (20.0, 22.0) |
| Oxygen saturation (%) | 94.0  (87.5, 98.0) | 99.0  (96.0, 100.0) | 98.0  (97.0, 99.0) | 96.0  (90.5, 98.0) | 98.5  (97.0, 100.0) | 98.0  (97.0, 100.0) |

### Data indicate the mean (SD) or median (Q1, Q3)

### SBP: Systolic blood pressure; DBP: Diastolic blood pressure; MAP: Mean arterial pressure

### *Significantly different from the hour-0 in the standard-rate group (p = 0.04)

**Figure S1** Mean hourly intravenous fluid volume per body weight (ml/kg) during the 6-hour intervention period. The error bars represent the standard deviation.


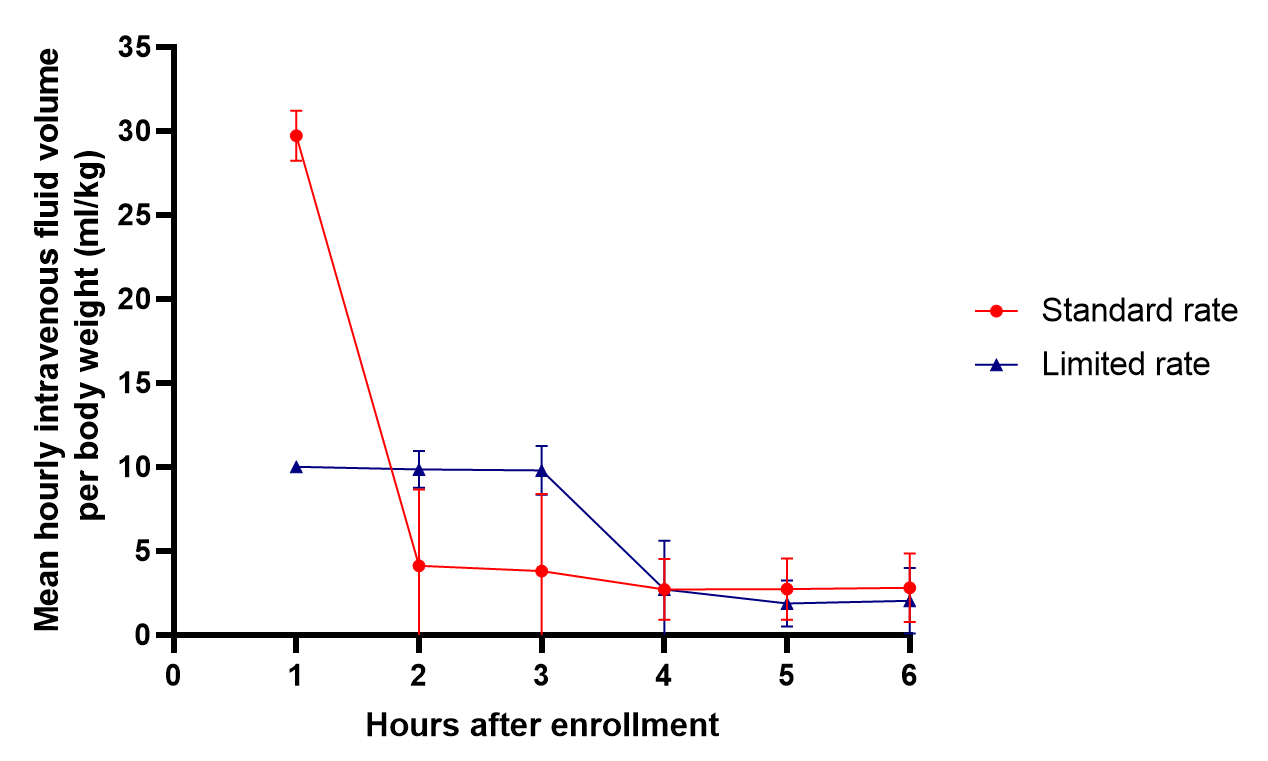


**Figure S2** Cumulative number of patients with the need of vasopressors during the 6-hour intervention period.


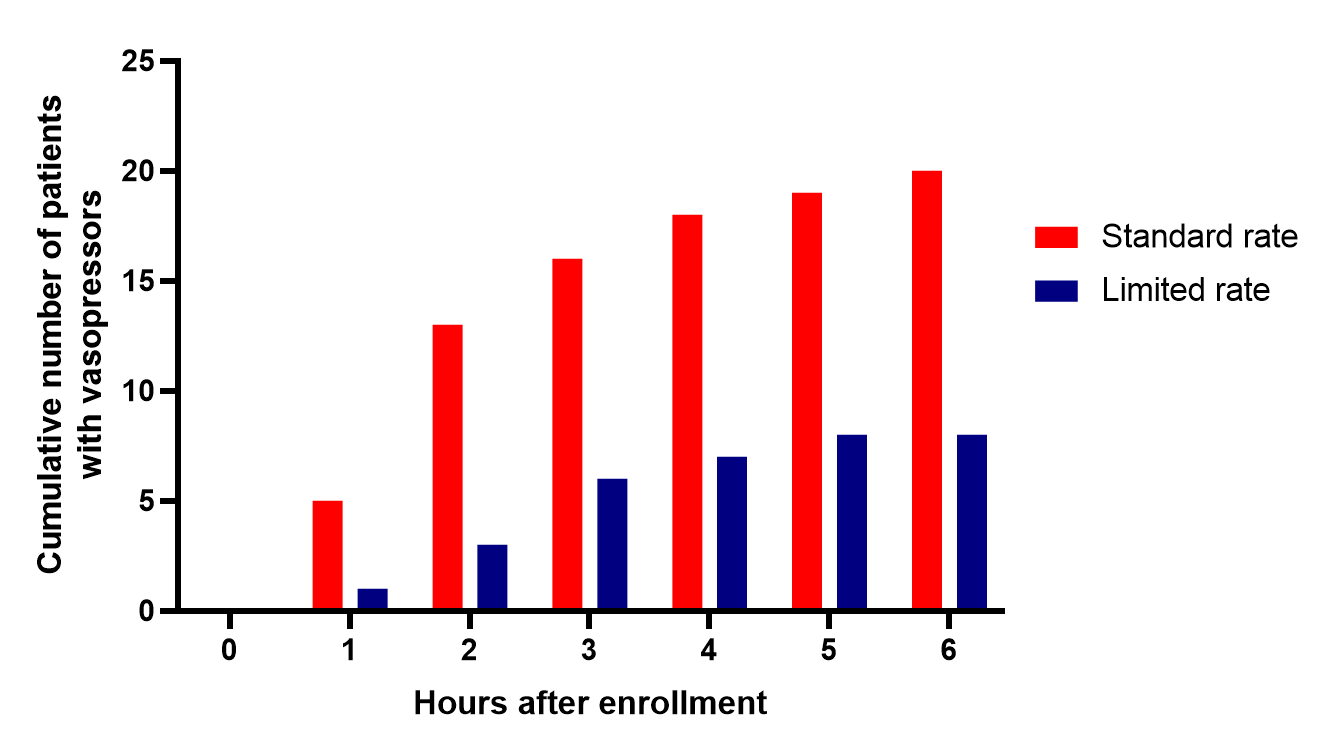


**Figure S3** Cumulative number of mechanically-ventilated patients during the 6-hour intervention period.


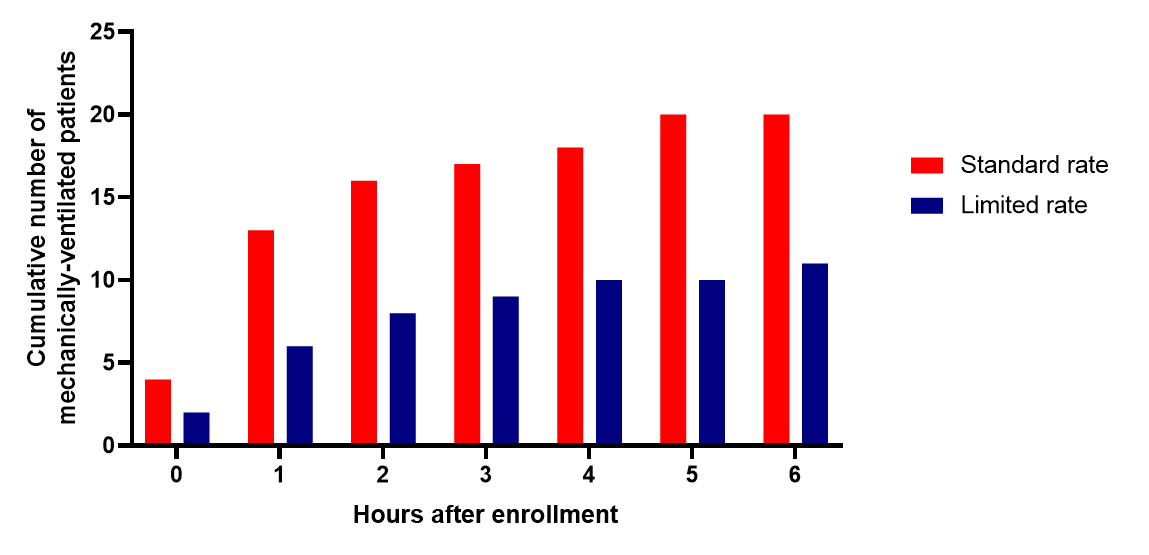

Supplement: Supplementary file 1 — Additional file 1: Table S1. Hemodynamic data of the patients during the intervention. Figure S1. Mean hourly intravenous fluid volume per body weight (ml/kg) during the 6-hour intervention period. The error bars represent the standard deviation. Figure S2. Cumulative number of patients with the need of vasopressors during the 6-hour intervention period. Figure S3. Cumulative number of mechanically-ventilated patients during the 6-hour intervention period. [file 40560_2020_515_MOESM1_ESM.doc]
